# Exposure to daily mean and maximum 1-hour PM_2.5_ concentrations and pediatric respiratory mortality in the Mexico City Metropolitan Area

**DOI:** 10.1097/EE9.0000000000000408

**Published:** 2025-06-25

**Authors:** Iván Gutiérrez-Avila, Robert O. Wright, María José Rosa, Allan C. Just

**Affiliations:** a Department of Environmental Medicine, Icahn School of Medicine at Mount Sinai, New York, New York; b Institute for Exposomic Research, Icahn School of Medicine at Mount Sinai, New York, New York; c Department of Epidemiology, School of Public Health, Brown University, Providence, Rhode Island; d Institute at Brown for Environment and Society, Brown University, Providence, Rhode Island

**Keywords:** PM_2.5_, Respiratory mortality, Infant mortality, Short-term exposure, Case-crossover study

## Abstract

**Background:**

Few studies have evaluated the association between short-term PM_2.5_ exposure and children’s respiratory mortality. This study examines the relationship between daily mean and maximum 1-hour PM_2.5_ exposures and age-specific pediatric respiratory mortality, addressing a gap in understanding the effects of subdaily PM_2.5_ peaks.

**Methods:**

We analyzed ICD-10-coded mortality records (n = 90,566) from the Mexico City Metropolitan Area (2004–2019). PM_2.5_ exposures came from our satellite-based models for daily mean and maximum 1-hour concentrations. Using a time-stratified case-crossover design and conditional logistic regression with distributed lags, we examined associations between PM_2.5_ and nonaccidental mortality, and specific respiratory conditions (e.g., influenza, pneumonia, bronchopulmonary dysplasia) across neonates, infants, children, and adolescents, accounting for sex-based effect modification. Our models included negative control exposures to address potential confounding.

**Results:**

Among all age groups, infants were the most affected by daily mean and maximum 1-hour PM_2.5_ concentrations. Mean PM_2.5_ was associated with higher risk of respiratory, and influenza and pneumonia mortality in infants. In the same age group, an increase of 10 μg/m^3^ in the maximum 1-hour PM_2.5_ concentration was associated with nonaccidental (odds ratio [OR][lag_0_] = 1.02 [95% confidence interval {CI}: 1.00, 1.03]), respiratory (OR[lag_0_] = 1.04 [95% CI: 1.02, 1.06]), influenza and pneumonia (OR[lag_0_] = 1.05 [95% CI: 1.02, 1.08]), and bronchopulmonary dysplasia-related (OR[lag_0_] = 1.07 [95% CI: 1.00, 1.15]) mortality. Our results suggest effect modification by sex in the association between mean PM_2.5_ and respiratory mortality, with positive associations observed primarily in male neonates and adolescents.

**Conclusions:**

Our study contributes to the evidence on the association between daily PM_2.5_ exposure and pediatric respiratory mortality, while also revealing new insights into the impact of maximum 1-hour PM_2.5_ on age- and cause-specific respiratory mortality.

**What this study adds**

This study provides critical insights into the effects of daily mean and maximum 1-hour PM_2.5_ exposures on age-specific pediatric respiratory mortality. Leveraging satellite-based PM_2.5_ estimates and a comprehensive official mortality dataset, it identifies infants as the most vulnerable group, facing heightened risks from short-term exposures. Sex-based differences suggest stronger respiratory mortality associations with mean PM_2.5_ for male neonates and adolescents. By analyzing both daily and subdaily peak pollution exposures, the study highlights the importance of improving air quality to protect children’s health and deepens understanding of acute impacts on vulnerable populations in moderately polluted urban areas.

## Background

Globally, respiratory diseases are a predominant cause of pediatric morbidity and mortality. However their distribution disproportionally affects low- and middle-income countries (LMICs), where it is estimated that over 95% of all deaths related to acute lower respiratory infections (approximately 1.3 million) occur each year.^1^ Despite the infectious nature of some respiratory diseases during childhood and adolescence, their exacerbation leading to death is determined by factors such as comorbidities, type of infection, socioeconomic status (SES), inadequate breastfeeding, malnutrition, health care access, and environmental exposures, including fine particulate matter (PM_2.5_).^2–4^ Over 93% of children and adolescents worldwide are exposed to PM_2.5_ levels exceeding World Health Organization (WHO) guidelines—about 630 million from LMICs in 2016—making PM_2.5_ a major risk factor for children’s respiratory health.^5–7^ Pediatric mortality is a crucial public health indicator, and reducing infant and child mortality is a core Sustainable Development Goal set by the UN General Assembly to prevent avoidable deaths in these age groups.^8,9^

Despite extensive research on the adverse effects of PM_2.5_ on children’s respiratory health—such as cough, wheezing, infection severity, asthma exacerbation, and reduced FVC—^10–13^ few studies have delved into the association between short-term PM_2.5_ exposure and respiratory mortality across pediatric age groups.^14–16^ A recent review and meta-analysis by Luben et al found short-term exposure to air pollutants (NO_2_, SO_2_, CO, PM_10_) consistently linked to postneonatal respiratory and sudden infant mortality. However, limited studies and methodological differences on PM_2.5_ and infant mortality prevented inclusion in meta-analyses, resulting in inconclusive findings.^17^ PM_2.5_ is known to cause more severe health effects than PM_10_ by reaching deeper lung tissue and inducing inflammation, while PM_10_ mainly deposits in the upper airways.^18^

Previous studies associate PM_2.5_ exposure with pediatric respiratory diseases in LMICs, including Mexico,^19–23^ but its link to respiratory mortality is less understood.^17^ Recently, focus has increased on studying subdaily peak PM_2.5_ exposures, especially as episodic extremes such as wildfire events from climate change become more frequent.^24^ However, evidence remains limited on the relationship between peak PM_2.5_ levels (e.g., daily maximum) and pediatric respiratory mortality. Although Mexico City’s air quality was once deemed highly dangerous for children’s health,^25^ it now reflects typical urban levels for LMICs.^26^ This study examines the association between pediatric respiratory mortality with daily mean and maximum 1-hour (max-1hr) exposures in the Mexico City Metropolitan Area while also exploring effect modification by sex.

## Methods

### Study design and population

We implemented a time-stratified case-crossover study design. Control days were matched to case days, aligning them with the same day of the week and within the same month and year as the corresponding case day—specifically, three or four control days per case. This method inherently controls for individual time-invariant confounders, long-term trends, and seasonal effects, ensuring unbiased estimates from conditional logistic regression.^27^ We obtained mortality records from the National Institute of Statistics and Geography of Mexico (INEGI) for the period from 1 February 2004 to 31 December 2019.

All mortality records included information on date of death, sex (“assigned at birth”), age, geographic identifiers for place of residence at the submunicipality level, and primary underlying cause of death classified according to the Tenth Revision of the International Classification of Diseases (ICD-10). We restricted our analysis to nonaccidental mortality (ICD-10 codes: A00-R99) and, within this broad category, we focused on deaths from respiratory outcomes—that is, the J group from the ICD-10. Within the category of total respiratory mortality (ICD-10 codes: J00-J99), we organized death records into mutually exclusive group-specific outcomes, including: influenza and pneumonia (ICD-10 codes: J09-J18), acute lower respiratory infections (ICD-10 codes: J20-J22), and chronic lower respiratory diseases (ICD-10 codes: J40-J47), and explored their association with PM_2.5_ exposures. We removed from our analyses death causes not likely to be influenced by PM_2.5_ exposure (ICD-10 codes: J69.0, J691, and J698 for aspiration pneumonia).^28^ We also evaluated the association between short-term exposure to PM_2.5_ with mortality from bronchopulmonary dysplasia (BPD) (ICD-10 code P27.1), as BPD is closely related to respiratory complications leading to infant mortality, especially in premature infants.^29^ Information about preterm or term delivery status, and comorbidities was not available. Mortality outcomes were classified into four pediatric age groups per American Academy of Pediatrics guidelines: infancy (neonatal: first 28 days, postneonatal: 28 days to 1 year), childhood (2–12 years), and adolescence (13–21 years).^30^

### Environmental exposures

We used daily mean and max-1hr PM_2.5_ estimates with a spatial resolution of 1 km × 1 km. These estimates came from our models developed for the Mexico City Metropolitan Area, employing extreme gradient boosting (XGBoost) and inverse-distance weighting, incorporating satellite-based aerosol optical depth data, meteorological information, and land-use variables.^31^ Our estimates for daily mean and max-1hr PM_2.5_ are available for the periods from 2004 to 2019 and from 2011 to 2019, respectively. Model performance was evaluated using leave-one-station-out cross-validation. The daily mean and max-1hr PM_2.5_ models achieved mean absolute errors of 3.68 μg/m^3^ (baseline median absolute deviation [MAD]: 8.55 μg/m^3^) and 9.20 μg/m^3^ (baseline MAD: 15.64 μg/m^3^), respectively, indicating strong predictive accuracy for both exposure metrics. While we reported additional metrics such as *R*^2^ and root mean square error for completeness, we emphasized mean absolute error as our primary metric due to its robustness to extreme values. Daily mean air temperature estimates with the same 1 km x 1 km resolution came from our satellite-based land surface temperature models for Central Mexico.^32^ Our analyses were confined to the predominantly urban Mexico City Metropolitan Area, where PM_2.5_ and temperature estimates were available. Exposure data were assigned to mortality records based on residential geographic identifiers within 561 submunicipal areas, or “localities,” as defined by INEGI. For 250 urban localities characterized by census-provided polygons, daily exposures were estimated through population-weighted aggregation, with population density data from the Gridded Population of the World (GPWv4) ~1-km raster cells.^33^ For the 361 rural localities, exposure to PM_2.5_ and temperature was assigned using the 1 km x 1 km grid cell containing the corresponding census-assigned point.

### Statistical analyses

**Figure 1.**
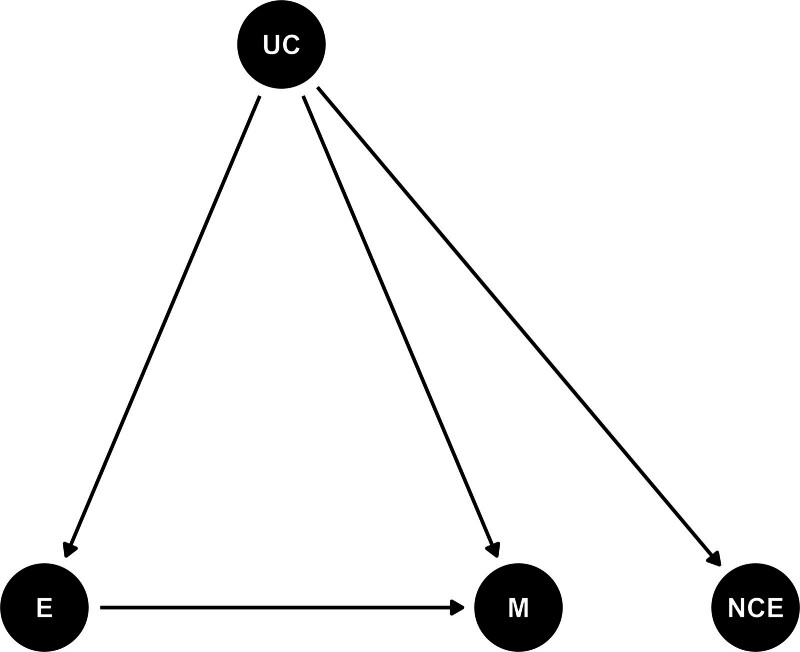
Directed acyclic graph representing the relationship between the negative control exposure (NCE) terms (i.e., 1-day lead mean and max-1hr PM_2.5_ concentrations), and potential unmeasured confounders (UC) in the association between the actual exposures (E) (i.e., daily mean and max-1h PM_2.5_) and mortality (M) outcomes.

The odds ratios (ORs) for all dependent variables associated with short-term exposure to PM_2.5_ were estimated with linear terms in stratified Cox proportional hazards models, equivalent to conditional logistic regression. To address the delayed effects of exposure to PM_2.5_ on all mortality outcomes we included distributed-lag terms up to 6 days before the case day to estimate mortality risks (as seven separate terms).^34^ We avoided extending lagged exposures beyond 6 days, to avoid autocorrelation in the overlapping exposure series of case and control days.^35,36^ All our models were adjusted for nonlinear effects of temperature with quadratic b-splines (4 degrees of freedom) with equally spaced knots.^37^ To assess potential residual confounding, we included negative control exposure (NCE) terms as 1-day lead PM_2.5_ (air pollution levels the day after a recorded death) in our regression models. While NCEs do not causally affect outcomes, they can share associations with unmeasured confounders similar to the actual exposure. Adjusting for 1-day lead PM_2.5_ helps account for confounding from unmeasured covariates. Figure 1 displays a directed acyclic graph illustrating this approach. By comparing primary exposure-outcome associations before and after NCE inclusion, we evaluated potential confounding. Minimal change in associations, alongside a null NCE-outcome association, indicates limited confounding.^38–40^

We evaluated season-specific associations between respiratory mortality and both PM_2.5_ exposures to assess potential variations across different times of the year, which are known to influence PM_2.5_ concentration levels. We used locally defined seasons observed in Mexico City, categorized as follows: hot season (March to May), rainy season (June to October), and cold season (November to February).^41^

We addressed potential effect modification in the associations between PM_2.5_ and respiratory mortality (ICD-10: J00-J99) with stratified analyses (subgroup analyses) by sex (males and females). The statistical analysis of the difference between two sex strata was conducted by calculating the 95% confidence interval (CI) of their difference in the log odds scale as follows:

95% CI = (E_1_−E_2_) ± 1.96√(σ_1_^2^+σ_2_^2^)

Where E_1_ and E_2_ are the coefficients of the association for strata 1 and 2, and σ_1_ and σ_2_ are the standard errors of these two groups.^42^ Finally, we explored whether the effect size of the associations between daily mean and max-1hr PM_2.5_ with respiratory and BPD mortality were statistically different by comparing their associations rescaled per unit increase of their respective MADs, and their Akaike Information Criterion (AIC) scores as a measure of goodness of fit.

For ease of interpretation and a direct comparison with previous studies, all associations are presented as ORs per each 10 μg/m^3^ higher PM_2.5_. All analyses were performed in R version 4.2.1.^43^ with packages: *data.table*,^44^ *survival*,^45^ *dlnm*,^46^ and *exactextractr*.^47^ All mortality records in our analyses are publicly available, and the senior author received an exempt human research determination (45 CFR 46.101(b), Category 4) from the Icahn School of Medicine at Mount Sinai IRB.

## Results

Table 1 describes the study population’s general characteristics. During the study period, we observed 90,566 nonaccidental deaths (ICD-10: A00-AR99) across pediatric age groups in the Mexico City Metropolitan Area. Respiratory mortality ranked fourth, after perinatal conditions (ICD-10: P00-P96), external causes (ICD-10: V01-Y98), and congenital diseases (ICD-10: Q00-Q99). Table S1; https://links.lww.com/EE/A359 shows a detailed ICD-10 breakdown. Our main analysis targeted 11,596 deaths from ICD-10 blocks J and P (12.8% of total nonaccidental deaths), including 10,928 respiratory and 668 perinatal deaths. Influenza and pneumonia (ICD-10: J09-J18) were the most common respiratory causes, while chronic lower respiratory diseases (ICD-10: J40-J47) were the least common. Overall, infant mortality comprised the largest proportion of total deaths (74.3%), while neonatal mortality accounted for the lowest (5.3%), with more deaths in males (57%) than in females (43%).

**Table 1.** Characteristics of the study population and environmental exposures in the Mexico City Metropolitan Area from 2004 to 2019

Variable	Frequency
Total nonaccidental deaths	90,566
(ICD-10 codes: A00-R99)	
Mortality groups analyzed (ICD-10 codes)	11,596 (100%)
Respiratory (Group J)	10,928 (94.2%)
Perinatal conditions (Group P)	668 (5.8%)
Cause-specific mortality (ICD-10-codes)	
Influenza and pneumonia (J09-J18)	6,128 (52.8%)
Other respiratory	2,143 (18.5%)
Lower respiratory infections (J20-J22)	2,063 (17.8%)
Bronchopulmonary dysplasia (P271)	668 (5.8%)
Chronic lower respiratory diseases (J40-J47)	594 (5.1%)
Age group	
Neonates (<1 month-old)	617 (5.3%)
Infants (1 month–1 year old)	8,614 (74.3%)
Children (2–12 years old)	1,317 (11.4%)
Adolescents (13–21 years old)	1,048 (9.0%)
Sex	
Male	6,612 (57%)
Female	4,984 (43%)
PM_2.5_ daily mean (µg/m^3^)	
Mean (SD)	26.6 (12.9)
Median (min, max)	25.1 (1.00, 180)
PM_2.5_ 1-hr max (µg/m^3^)	
Mean (SD)	49.2 (23.4)
Median (min, max)	46.3 (9.80, 281)
Temperature (Celsius)	
Mean (SD)	15.5 (2.81)
Median (min, max)	15.6 (2.00, 23.6)

Max-1hr PM_2.5_ was only available for the period from 2011 to 2019.

Throughout the study period, a minimum of 71% of observed days per year surpassed the most recent version of the WHO guidelines for PM_2.5_ issued in 2021, which recommend a limit of 15 μg/m^3^ in a 24-hour average. Likewise, the annual WHO guideline of 5 μg/m^3^ was exceeded by at least four-fold each year. Currently, there is no WHO guideline recommended for subdaily PM_2.5_ levels, such as the max-1hr PM_2.5_ concentration; for which a range of 9.8–281 μg/m^3^ was observed from 2011 to 2019. Figure S1; https://links.lww.com/EE/A359 shows the time series of daily mean and max-1hr PM_2.5_ concentrations for 2011 and 2019 compared with the 24-hour average WHO recommended limit.

### Associations with daily mean PM_2.5_

**Figure 2.**
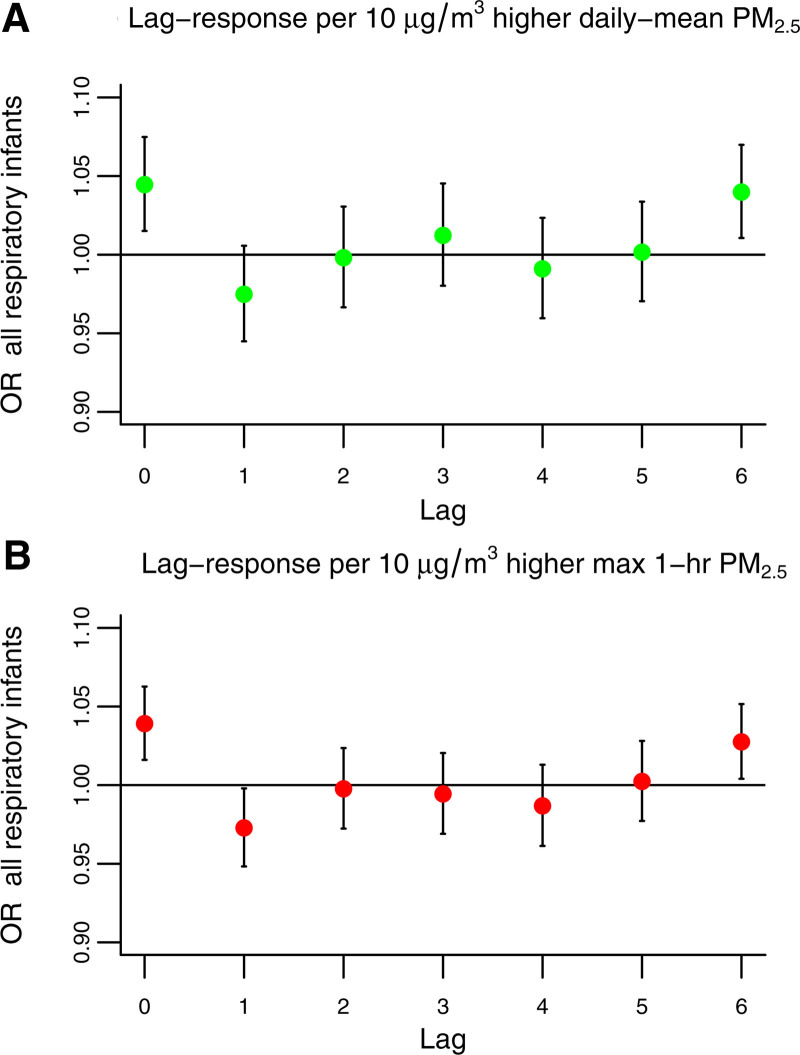
Lag-response plots for daily mean (2004–2019) and max-1hr (2011–2019) exposure to PM_2.5_ with respiratory mortality (ICD10 codes: J00-J99) in the Mexico City Metropolitan Area.

Overall, exposure to daily mean PM_2.5_ was not associated with nonaccidental mortality in any of the four age groups analyzed. For respiratory mortality, same-day exposure to 10 μg/m^3^ higher PM_2.5_ was associated with infant respiratory mortality with OR(lag_0_) = 1.05 (95% CI: 1.02, 1.08), as shown in Figure 2A; with a cumulative association over seven days of OR(lag_06_) = 1.06 (95% CI: 1.01, 1.11). Among all respiratory outcomes (Figure S2; https://links.lww.com/EE/A359), the largest association was observed for daily mean PM_2.5_ with influenza and pneumonia in infants with the same-day association of OR(lag_0_) = 1.07 (95% CI: 1.04, 1.12), 2-day cumulative association of OR(lag_01_) = 1.06 (95% CI: 1.01, 1.11), and 1-week cumulative association of OR(lag_06_) = 1.11 (95% CI: 1.05, 1.19) per 10 μg/m^3^ higher PM_2.5_. Cumulative associations over 6 and 3 days were also observed for chronic lower respiratory mortality in infants OR(lag_05_) =1.35 (95% CI: 1.03, 1.78), and acute lower respiratory infections in adolescents OR(lag_02_) = 3.00 (95% CI: 1.30, 6.50), respectively. BPD mortality was not associated with daily mean PM_2.5_ exposure.

### Associations with max-1hr PM_2.5_

Exposure to max-1hr PM_2.5_ was associated with nonaccidental mortality in infants (OR[lag_0_] = 1.02 [95% CI: 1.00, 1.03]), and with some suggestive evidence for associations at later lags in the children age group (Figure S3; https://links.lww.com/EE/A359). The max-1hr PM_2.5_ concentration was also associated with total respiratory mortality in infants (OR[lag_0_]) = 1.04 [95% CI: 1.02, 1.06]) as shown in Figure 2B, and children (OR[lag_3_] = 1.08 [95% CI: 1.02, 1.14]) (Figure S4; https://links.lww.com/EE/A359). Among all group-specific respiratory mortality outcomes, influenza and pneumonia (infants OR[lag_0_]= 1.05 [95% CI: 1.02, 1.08], and children OR[lag_3_] = 1.10 [95% CI: 1.03, 1.18]), and chronic lower respiratory diseases (children OR[lag_3_] = 1.53 [95% CI: 1.08, 2.17], and adolescents OR[lag_3_] = 1.28 [95% CI: 1.04, 1.57]) were also associated with max-1hr PM_2.5_ (Figure S5; https://links.lww.com/EE/A359).

**Figure 3.**
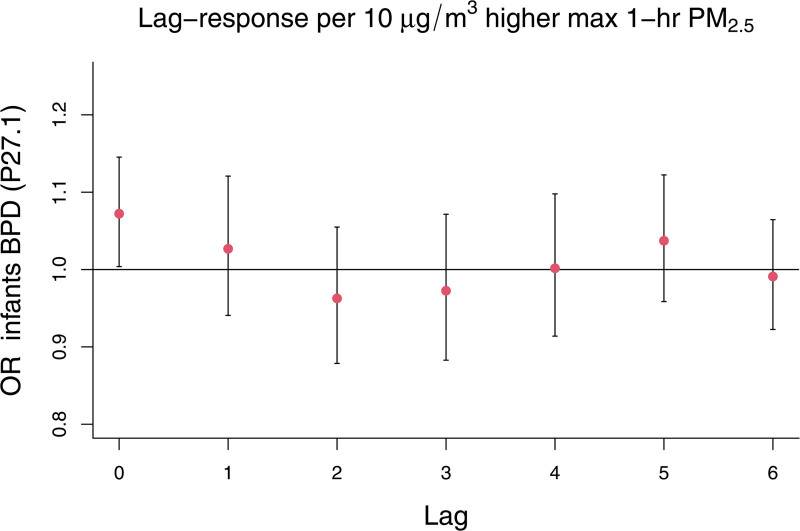
Lag-response association for exposure to daily max-1hr PM_2.5_ with mortality for bronchopulmonary dysplasia (ICD-10 code P27.1) in the Mexico City Metropolitan Area (2011–2019).

BPD mortality (Figure 3) showed a same day association of OR(lag_0_) = 1.07 (95% CI: 1.00, 1.15), and a 2-day cumulative association of OR(lag_01_) = 1.10 (95% CI: 1.00, 1.21) per 10 μg/m^3^ higher max-1hr PM_2.5_.

Overall, the inclusion of NCE terms (PM_2.5_-lead_1_) in our models, had minimal effects on the associations between mean and max-1hr PM_2.5_ with respiratory mortality, and those terms were not associated with any mortality outcome as observed in Table 2 for the infant age group.

**Table 2.** Infant mortality associations as ORs and 95% CIs per 10 μg/m^3^ higher PM_2.5_ on the event day (lag_0_) and 1 day after used as negative control exposure (PM_2.5_-lead_1_)

Exposure metric/outcome	Same day exposure	Negative control exposure^c^
	(PM_2.5_–lag0)	(PM_2.5_–lead1)
	OR (95% CI)	OR (95% CI)
Daily-mean PM_2.5_		
Respiratory^a^	1.04 (1.02, 1.07)	1.00 (0.97, 1.03)
^b^	1.05 (1.02, 1.07)	
Influenza and pneumonia^a^	1.07 (1.04, 1.11)	0.98 (0.94, 1.02)
^b^	1.07 (1.03, 1.10)	
Max 1-hr PM_2.5_		
Nonexternal^a^	1.01 (1.00, 1.03)	1.01 (0.99, 1.04)
^b^	1.02 (1.01, 1.03)	
Respiratory^a^	1.04 (1.02, 1.06)	0.97 (0.92, 1.02)
^b^	1.04 (1.01, 1.06)	
Influenza and pneumonia^a^	1.05 (1.02, 1.08)	0.97 (0.90, 1.03)
^b^	1.05 (1.02, 1.08)	
Bronchopulmonary dysplasia^a^	1.07 (1.00, 1.15)	1.06 (0.91, 1.23)
^b^	1.08 (1.01, 1.15)	

a Association after inclusion of negative control exposure.

b Association before inclusion of negative control exposure.

c Association with negative control exposure.

**Figure 4.**
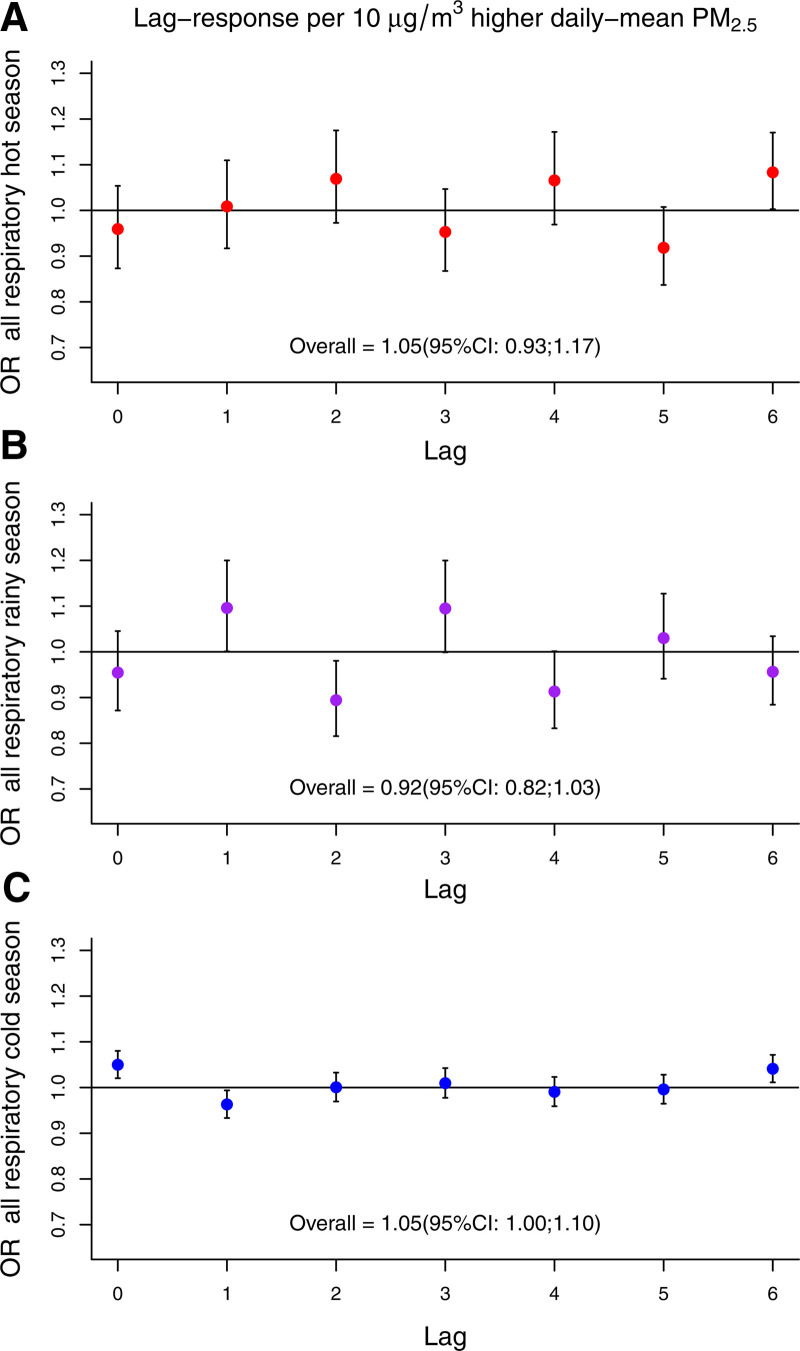
Season-specific lag-response plots for short-term exposure to PM_2.5_ with respiratory mortality in the Mexico City Metropolitan Area (2004–2019). Hot season: March–May; Rainy season: June–October; and Cold season: November–February.

Seasonal analysis showed daily mean PM_2.5_ associated with respiratory mortality across all seasons, though with varying temporal patterns. Immediate and precise effects were most notable in the cold season (Figure 4), with max-1hr PM_2.5_ showing a similar trend only in this season (Figure S6; https://links.lww.com/EE/A359).

Sex-specific associations between mean PM_2.5_ and respiratory mortality were mainly observed in males, with positive associations in neonates (OR[lag_0_] = 1.21 [95% CI: 1.01, 1.45]) and infants (OR[lag_0_] = 1.07 [95% CI: 1.03, 1.11]) (Figure S7; https://links.lww.com/EE/A359). Cumulative associations were also observed in female infants (OR[lag_06_] = 1.08 [95% CI: 1.00, 1.16]). Some evidence of sex-based effect modification was observed in neonates and adolescents, with positive associations in males at specific cumulative exposures (Table S2; https://links.lww.com/EE/A359). Analyses comparing associations of daily mean and max-1hr PM_2.5_ with respiratory and BPD mortality (February 2011–December 2019) showed no significant difference in effect sizes, though AIC indicated a slightly better model fit for max-1hr PM_2.5_ (Table S3; https://links.lww.com/EE/A359).

## Discussion

This study evaluated the associations of daily mean (2004–2019) and max-1hr PM_2.5_ (2011–2019) with nonaccidental and pediatric respiratory mortality in the Mexico City Metropolitan Area. Both PM_2.5_ metrics showed positive associations with mortality across all pediatric age groups, with the most consistent associations in infants. Max-1hr PM_2.5_ was linked to a broader range of outcomes than mean PM_2.5_. Our results align with limited prior studies on short-term PM_2.5_ exposure and pediatric respiratory mortality,^14,15,48^ and those focused on PM_10_ and infant mortality.^17^

Unlike other studies in megacities that used limited PM_2.5_ data from a few ground stations, assuming uniform exposure across large areas,^15,48^ we used advanced methods to predict highly spatially resolved PM_2.5_ concentrations for the Mexico City Metropolitan Area. Most research on PM_2.5_ health effects has relied on 24-hour averages (daily mean PM_2.5_).^49^ Emerging evidence, however, indicates that subdaily peak exposures may pose even higher risks, particularly for vulnerable populations, though few studies have explored this for pediatric mortality.^50^ Therefore, we also included the max-1hr PM_2.5_ concentration as an exposure metric in our analyses, under the premise that peak exposures can also trigger acute pediatric mortality.^49^ In children, for instance, some evidence has shown higher associations of asthma symptoms from exposure to the max-1hr and 8hr PM_10_ averages compared with the daily mean.^51^ In our study, associations between daily mean and max-1hr PM_2.5_ with respiratory and BPD mortality were similar (Table S3; https://links.lww.com/EE/A359); however, max-1hr PM_2.5_ slightly improved models fit based on AIC. This finding suggests a need for further research on subdaily exposures, especially regarding episodic peaks from increasingly frequent wildfires linked to climate change. Understanding these extreme, short-term pollution events is an emerging priority in air pollution epidemiology.^52,53^

Older local studies in the Mexico City region have reported positive associations between PM exposure and infant mortality. Loomis et al^16^ found a 6.9% (95% CI: 2.5, 11.3) increase in nonaccidental infant mortality risk per 10 μg/m^3^ higher daily mean PM_2.5_ (lags 3–5) for 1993–1995. Using citywide PM_2.5_ averages from 14 municipalities, Carbajal Arroyo et al^54^ reported associations between daily mean PM_10_ with nonaccidental (OR[lag_02_] = 1.063 [95% CI: 1.001, 1.132]) and respiratory (OR[lag_2_] = 1.098 [1.021, 1.180]) infant mortality for 1997–2005. While limited in number, other international studies support our findings: in Tokyo, Yorifuji et al^15^ observed positive associations between same-day PM_2.5_ and all-cause postneonatal (OR[lag_0_] = 1.10 [95% CI: 1.02, 1.19]) and total infant respiratory mortality (OR[lag_0_] = 1.30 [95% CI: 1.01, 1.67]). In a national Chinese study, He et al^14^ found that a 10 μg/m^3^ increase in PM_2.5_ (2-day average, lag_0–1_) increased all-cause mortality by 1.15% (95% CI: 0.65%, 1.65%) and pneumonia-related mortality by 1.25% (95% CI: 0.01%, 2.51%) in children under 5 years old.

Although our study did not specifically examine subchronic (monthly) PM_2.5_ exposure, our findings align with Wang et al’s^48^ results for Beijing, which also showed larger mortality associations for infants compared with older children. In California, Woodruff et al^28^ reported a higher risk of infant respiratory mortality (OR = 2.13, 95% CI: 1.12, 4.05) per 10 μg/m^3^ increase in PM_2.5_ averaged over the life period of the deceased, with larger associations among low birth weight infants and those with BPD. In Brazil, Braga et al^55^ found infants and adolescents most susceptible to hospital admissions for respiratory issues following short-term PM_10_ exposure (7-day cumulative effects), reporting a 9.4% (95% CI: 7.9, 10.9) increased risk for infants and 5.1% (95% CI: 0.3, 9.8) for adolescents per 35 μg/m^3^ increase in PM_10_.

Overall, our results showed consistent associations between both mean and max-1hr PM_2.5_ exposures with infant respiratory mortality (Table 2). Infants’ heightened susceptibility may stem from their immature immune systems, slower detoxification processes, and higher respiratory rates, including more mouth breathing, which increases PM_2.5_ inhalation, facilitating the movement of particles into the lungs and increasing the received dose. Additionally, infants’ breathing near ground level, where traffic pollutants concentrate, raises their particle exposure.^56–59^ These characteristics, along with preexisting health conditions, may increase susceptibility of infants and children to the lagged and cumulative associations observed after PM_2.5_ exposure.^14^ Rapid physiological changes during infancy and childhood underscore the need for targeted studies on these age groups. Associations between short-term PM_2.5_ exposure and mortality in adolescents are rarely studied independently and are often grouped with children or young adults, complicating comparisons. Extrapolating adult findings to early-life environmental exposures is generally discouraged.^56^

Although more evidence on PM_2.5_-related mortality in this age group is needed to support our findings, a South African study reported higher odds of respiratory diseases (e.g., allergies, wheezing, bronchitis, and asthma) among adolescents living in areas with high PM_2.5_ levels.^60^ Lung function develops linearly until adolescence, then diverges with sex differences. Therefore, it is essential to report age- and sex-specific air pollution effects in adolescents separately from those of younger children and adults to guide tailored health interventions during this transitional period of life.^61^

We found positive associations between PM_2.5_ exposure and respiratory mortality in all seasons, with the cold season showing the most precise associations. This may be related to the higher sample size in the number of deaths observed during the cold season (n = 5,624), compared with the hot (n = 2,050) and rainy (n = 3,254) seasons. Alternative explanations include a higher frequency of thermal inversion in the Valley of Mexico during this season, leading to a higher accumulation of PM_2.5_ and less variation in the observed PM_2.5_ concentrations, consequently reducing the potential of exposure misclassification in the cold season.^41^ Seasonal PM_2.5_ composition and a higher prevalence of respiratory infections during the cold season may also increase PM_2.5_ toxicity and health risks.^62,63^

Our stratified analyses looking at effect modification by sex showed that sex seemed to modify the association between short-term exposure to PM_2.5_ with respiratory mortality (Table S2; https://links.lww.com/EE/A359) in neonates and adolescents. Previous evidence also has reported higher frequency and severity of acute lower respiratory infections in males compared with females, which could be explained by faster biological development in females compared with males.^64^ Differential maturation in lung development of males relative to females may predispose male infants to childhood respiratory diseases.^65,66^ Surfactant, a crucial compound facilitating proper lung function, is produced earlier in females than in males, enabling a faster lung maturation process.^67^

This study has several strengths. We utilized an official national mortality dataset with information on the total number of deaths occurring in the Mexico City Metropolitan Area, therefore our results are representative of the study region and reflect the variation in exposure to PM_2.5_. Compared with previous time-series studies performed in the Mexico City Metropolitan Area, the case-crossover study design allowed us to analyze individual health records and assign exposures at the submunicipal level (i.e,. the smallest spatial unit on place of residence available in Mexican mortality records) instead of spatially aggregated daily counts, reducing the likely magnitude of exposure measurement error from the two exposure metrics included in our study. Also, the case-crossover study design inherently addresses time-invariant individual-level confounders, reducing the chance of bias in our findings. As shown in our results, the NCE terms (PM_2.5_-lead_1_ terms) included in our models were not associated with mortality outcomes; and after their inclusion, only small changes in the effect size of the associations were observed. Therefore, providing evidence that no relevant unmeasured confounders were omitted, and strengthening causal identification.^39^ Our study also has some limitations. First, the absence of information on preterm delivery and comorbidities, including congenital conditions, represents a notable gap, as these factors have been linked to higher infant respiratory mortality rates.^68^ Individual-level data on maternal education and other SES-related factors, such as nutritional status, housing conditions, health care access, and exposure to secondhand smoke at home, were not available. While the case-crossover study design inherently controls for such time-invariant factors, the lack of detailed information about other SES-related variables precluded the exploration of potential effect modification from those characteristics to identify vulnerable subgroups. Additionally, although we employed highly spatially resolved PM_2.5_ predictions for exposure assignment, the potential for exposure misclassification to PM_2.5_ cannot be ruled out due to modeling errors and the absence of information on time-location activity patterns across multiple microenvironments. Even in models mutually adjusted for daily mean and max-1hr PM_2.5_ concentrations, disentangling their independent effects remains challenging due to their inherent correlation and shared origin within the exposure model. More robust separation of subdaily and daily cumulative exposure effects—particularly when relying on daily-mean averages—would require additional temporal information, such as the exact time of death or, ideally, symptom onset, to better align exposure windows with relevant health events.^69^ On the exposure side, future studies leveraging high-temporal-resolution data from geostationary satellites, which provide multiple daytime aerosol-related atmospheric observations, could help address this limitation and improve our understanding of subdaily PM_2.5_ exposure dynamics and their health impacts.

## Conclusions

Daily mean and max-1hr PM_2.5_ concentrations were positively associated with pediatric respiratory mortality outcomes in the Mexico City Metropolitan Area, a region with moderate PM_2.5_ pollution levels. Among all pediatric age groups examined, infant mortality was most consistently associated with both PM_2.5_ exposure metrics. Furthermore, exposure to max-1hr PM_2.5_ was also associated with nonaccidental and BPD-related mortality. Emerging evidence suggests that adverse effects from peak exposures at subdaily time frames may increase the risk of acute mortality in vulnerable population segments, making our study particularly valuable.

## Conflicts of interest statement

The authors declare that they have no conflicts of interest with regard to the content of this report.

## Supplementary Material


