# Supplementary material for: Exposure to daily mean and maximum 1-hour PM2.5 concentrations and pediatric respiratory mortality in the Mexico City Metropolitan Area
Source: Environ Epidemiol. 2025 Jun 25;9(4):e408. doi: 10.1097/EE9.0000000000000408 (PMC12200233; doi:10.1097/EE9.0000000000000408)

Supplemental material

Table S1. Classification of causes of death according to ICD-10 across all pediatric age groups in the Mexico City Metropolitan Area during the period 2004-2019.

Table S2. Differences in log odds ratios for the cumulative association between mean $PM_{2.5}$ and respiratory mortality stratified by sex and age group in the Mexico City Metropolitan Area (2004-2019).

Table S3. Differences in log odds ratios of the cumulative association between daily mean and max-1hr $PM_{2.5}$ with respiratory and bronchopulmonary dysplasia mortality in the Mexico City Metropolitan Area (2011-2019)

Figure S1. Time-series of the daily mean and max-1hr concentrations of PM2.5 in the Mexico City Metropolitan Area

Figure S2. Lag - response plots for mean $PM_{2.5}$ and group-specific respiratory mortality in the Mexico City Metropolitan Area (2004-2019) for infants, children and adolescents.

Figure S3. Lag - response plots for max-1hr $PM_{2.5}$ and non-accidental mortality in the Mexico City Metropolitan Area (2011-2019) stratified by age group.

Figure S4. Lag - response plots for max-1hr $PM_{2.5}$ and respiratory mortality in the Mexico City Metropolitan Area (2011-2019) stratified by age group.

Figure S5. Lag - response plots for max-1hr $PM_{2.5}$ and group-specific respiratory mortality in the Mexico City Metropolitan Area (2011-2019) for infants and children.

Figure S6. Lag - response plots for max-1hr $PM_{2.5}$ and respiratory mortality in the Mexico City Metropolitan Area (2004-2019) stratified by season.

Figure S7. Lag -response plots between mean $PM_{2.5}$ exposure with respiratory mortality in the Mexico City Metropolitan Area (2004 - 2019), stratified by sex and age group.

Table S1. Classification of causes of death according to ICD-10 across all pediatric age groups in the Mexico City Metropolitan Area during the period 2004-2019.

ICD-10 Title (Block codes)	Frequency (%) Overall = 117,286 (100%)
Conditions Originating In The Perinatal Period (P00-P96)	28,548 (24.34%)
External Causes Of Morbidity And Mortality (V01-Y98)	25,774 (21.98%)
Congenital Malformations, Deformations And Chromosomal Abnormalities (Q00-Q99)	17,856 (15.22%)
Respiratory (J00-J99)	11,261 (9.60%)
Neoplasms (C00-D48)	7,235 (6.17%)
Infectious And Parasitic (A00-B99)	5,619 (4.79%)
Nervous (G00-G99)	5,020 (4.28%)
Circulatory (I00-I99)	3,745 (3.19%)
Endocrine, Nutritional And Metabolic (E00-E90)	3,212 (2.74%)
Digestive (K00-K93)	3,038 (2.59%)
Genitourinary (N00-N99)	2,347 (2.00%)
Blood And Certain Disorders Involving The Immune Mechanism (D50-D89)	1,265 (1.08%)
Symptoms, Signs And Abnormal Clinical And Laboratory Findings (R00-R99)	1,053 (0.90%)
Musculoskeletal System And Connective Tissue (M00-M99)	572 (0.49%)
Pregnancy (O00-O99)	496 (0.42%)
Mental (F00-F99)	142 (0.12%)
Skin And Subcutaneous Tissue (L00-L99)	83 (0.07%)
Ear And Mastoid Process (H60-H95)	13 (0.01%)
Eye And Adnexa (H00-H59)	7 (0.01%)

Table S2. Differences in odds ratios for the cumulative association between mean $PM_{2.5}$ and respiratory mortality stratified by sex and age-group in the Mexico City Metropolitan Area (2004-2019).

Group	lag	ORcum males	LLcum	ULcum	ORcum females	LLcum	ULcum	Difference (95%CI)[a]
Male neonates vs Female neonates	0	1.210	1.013	1.445	0.768	0.613	0.962	1.576 (1.183, 2.099)
	1	1.210	0.988	1.483	0.826	0.645	1.056	1.465 (1.064, 2.016)
	2	1.158	0.943	1.423	0.669	0.507	0.883	1.731 (1.225, 2.445)
	3	1.025	0.803	1.309	0.775	0.582	1.032	1.323 (0.908, 1.927)
	4	0.995	0.769	1.287	0.751	0.552	1.020	1.325 (0.887, 1.978)
	5	0.873	0.657	1.160	0.683	0.503	0.926	1.278 (0.842, 1.940)
	6	0.871	0.665	1.141	0.717	0.523	0.984	1.215 (0.802, 1.841)
Male infants vs Female infants	0	1.068	1.028	1.110	1.015	0.972	1.061	1.052 (0.993, 1.115)
	1	1.013	0.968	1.061	1.025	0.972	1.080	0.988 (0.922, 1.060)
	2	1.034	0.982	1.089	0.992	0.934	1.054	1.042 (0.963, 1.129)
	3	1.032	0.975	1.092	1.024	0.959	1.094	1.008 (0.924, 1.099)
	4	1.038	0.977	1.101	0.992	0.925	1.064	1.046 (0.954, 1.147)
	5	1.025	0.962	1.092	1.015	0.943	1.091	1.010 (0.917, 1.112)
	6	1.048	0.984	1.117	1.081	1.004	1.164	0.969 (0.880, 1.069)
Male children vs Female children	0	0.912	0.796	1.046	0.940	0.817	1.082	0.970 (0.798, 1.180)
	1	0.943	0.818	1.088	0.920	0.793	1.066	1.025 (0.835, 1.259)
	2	0.899	0.765	1.056	0.907	0.774	1.064	0.991 (0.790, 1.243)
	3	0.979	0.824	1.164	0.983	0.828	1.168	0.996 (0.780, 1.271)
	4	0.986	0.826	1.177	0.890	0.741	1.069	1.108 (0.859, 1.429)
	5	1.036	0.862	1.246	0.908	0.749	1.102	1.141 (0.874, 1.490)
	6	1.053	0.875	1.267	0.942	0.778	1.140	1.118 (0.857, 1.458)
Male adolescents vs Female adolescents	0	1.138	0.981	1.319	0.995	0.842	1.177	1.144 (0.915, 1.430)
	1	1.070	0.914	1.252	0.932	0.782	1.111	1.148 (0.907, 1.453)
	2	1.147	0.962	1.368	0.904	0.740	1.106	1.269 (0.971, 1.657)
	3	1.242	1.032	1.496	0.827	0.666	1.026	1.502 (1.130, 1.997)
	4	1.278	1.046	1.562	0.867	0.684	1.098	1.474 (1.081, 2.010)
	5	1.045	0.846	1.291	0.782	0.617	0.990	1.336 (0.973, 1.835)
	6	1.086	0.881	1.338	0.866	0.686	1.094	1.254 (0.917, 1.715)

Table S3. Differences in log odds ratios of the cumulative association between daily mean and max-1hr $PM_{2.5}$ with respiratory and BPD related mortality in the Mexico City Metropolitan Area (2011-2019)

Group	lag	Daily mean $PM_{2.5}$ OR (95%CI) OR cumulative	Lower Limit cumulative	Upper Limit cumulaive	Maximum 1hr $PM_{2.5}$ OR (95%CI) OR cumulative	Lower Limit cumulative	Upper Limit cumulative	Effect size difference (95% CI)	AIC mean $PM_{2.5}$	AIC max 1hr $PM_{2.5}$
All Respiratory Neonates	0	0.965	0.773	1.206	0.979	0.836	1.147	0.986 (0.750, 1.296)	623.16	620.25
	1	0.983	0.764	1.265	0.948	0.775	1.159	1.037 (0.751, 1.432)		
	2	0.915	0.679	1.234	0.834	0.644	1.080	1.098 (0.740, 1.630)		
	3	0.899	0.656	1.230	0.758	0.572	1.005	1.186 (0.777, 1.808)		
	4	0.774	0.559	1.073	0.703	0.521	0.948	1.102 (0.708, 1.715)		
	5	0.727	0.520	1.017	0.631	0.458	0.868	1.153 (0.725, 1.832)		
	6	0.643	0.457	0.905	0.584	0.420	0.813	1.102 (0.685, 1.772)		
All Respiratory Infants	0	1.082	1.028	1.138	1.064	1.026	1.104	1.016 (0.954, 1.082)	8319.07	8311.91
	1	1.024	0.963	1.089	1.018	0.969	1.069	1.007 (0.930, 1.089)		
	2	1.033	0.965	1.105	1.014	0.957	1.073	1.018 (0.932, 1.113)		
	3	1.021	0.949	1.100	1.005	0.943	1.071	1.017 (0.922, 1.121)		
	4	0.994	0.919	1.075	0.983	0.917	1.054	1.011 (0.910, 1.123)		
	5	0.987	0.908	1.072	0.987	0.916	1.064	1.000 (0.894, 1.118)		
	6	1.020	0.939	1.109	1.031	0.955	1.114	0.989 (0.883, 1.109)		
All Respiratory Children	0	0.855	0.737	0.993	0.932	0.841	1.032	0.918 (0.766, 1.100)	1772.69	1772.68
	1	0.866	0.744	1.006	0.898	0.796	1.013	0.964 (0.795, 1.169)		
	2	0.858	0.724	1.016	0.897	0.782	1.029	0.956 (0.769, 1.188)		
	3	0.990	0.828	1.182	1.009	0.872	1.167	0.981 (0.780, 1.235)		
	4	0.892	0.741	1.075	0.960	0.820	1.123	0.930 (0.729, 1.186)		
	5	0.929	0.768	1.125	0.989	0.837	1.168	0.940 (0.730, 1.211)		
	6	0.944	0.782	1.140	0.995	0.839	1.180	0.949 (0.736, 1.224)		
All Respiratory Adolescents	0	1.013	0.880	1.166	0.990	0.890	1.101	1.024 (0.858, 1.221)	1750.69	1751.34
	1	0.995	0.857	1.155	1.009	0.893	1.140	0.986 (0.813, 1.196)		
	2	0.988	0.836	1.167	0.966	0.840	1.111	1.022 (0.823, 1.271)		
	3	1.058	0.885	1.263	1.011	0.867	1.178	1.046 (0.827, 1.323)		
	4	1.075	0.885	1.307	1.047	0.888	1.234	1.027 (0.796, 1.326)		
	5	0.932	0.765	1.136	0.934	0.779	1.119	0.998 (0.764, 1.305)		
	6	0.913	0.751	1.110	0.935	0.779	1.123	0.976 (0.747, 1.275)		
Bronchopulmonary Dysplasia Infants	0	1.106	0.940	1.303	1.118	1.006	1.242	0.990 (0.815, 1.202)	1038.94	1036.14
	1	1.152	0.948	1.400	1.166	1.001	1.359	0.988 (0.771, 1.265)		
	2	1.061	0.868	1.298	1.098	0.924	1.303	0.967 (0.742, 1.260)		
	3	1.103	0.885	1.376	1.050	0.867	1.271	1.051 (0.785, 1.407)		
	4	1.029	0.814	1.300	1.052	0.854	1.298	0.978 (0.714, 1.338)		
	5	1.134	0.892	1.442	1.116	0.894	1.393	1.017 (0.733, 1.410)		
	6	1.105	0.865	1.412	1.100	0.875	1.383	1.005 (0.719, 1.405)		

The Odds Ratios and 95%CI are shown for a unit increase in the Median Absolute Deviations (MAD) of daily mean $PM_{2.5}$ or 1-hr max $PM_{2.5}$ exposures, respectively

Figure S1. Time-series of the daily mean and max-1hr concentrations of $PM_{2.5}$ in the Mexico City Metropolitan Area

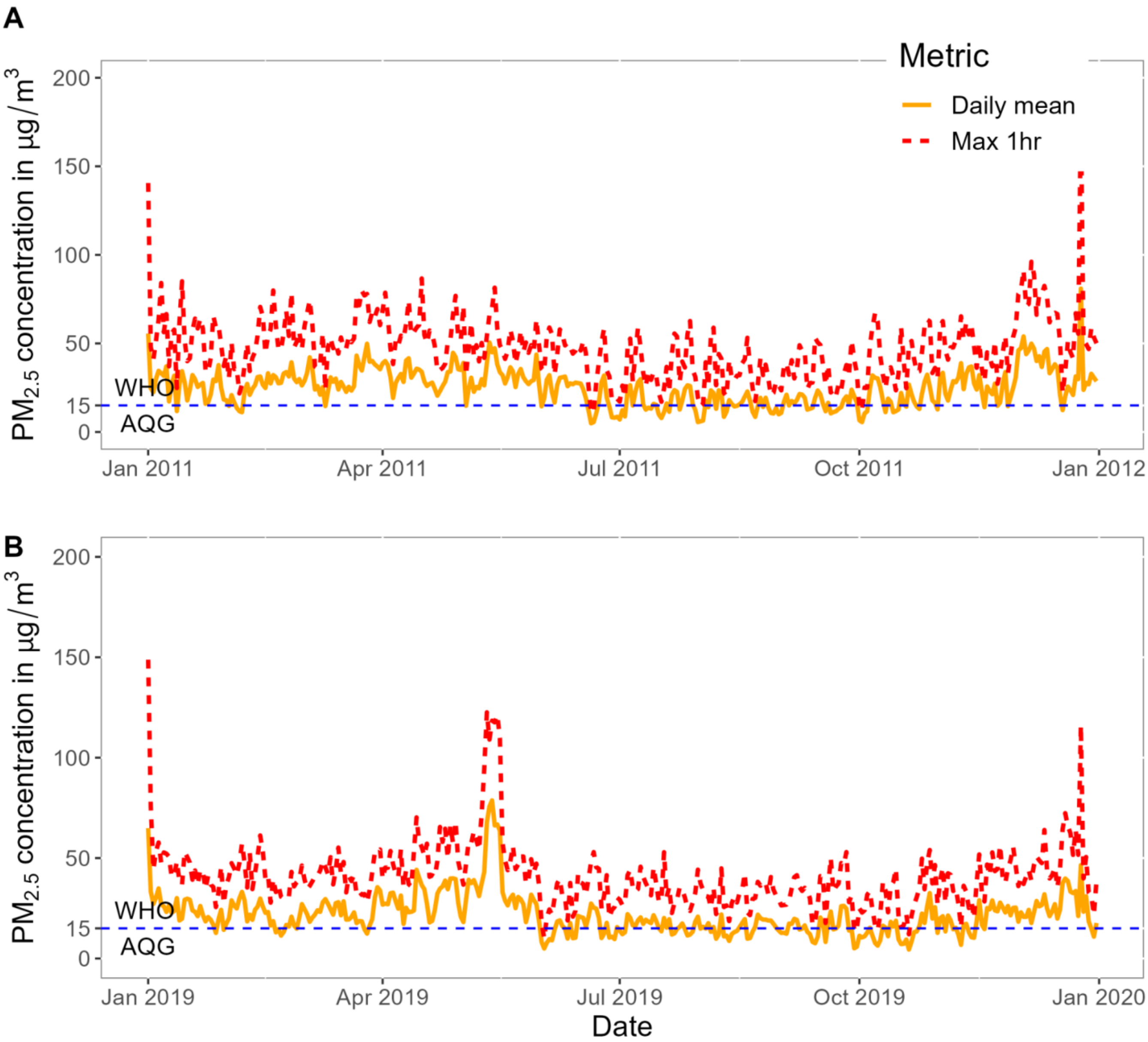

Figure S2. Lag - response plots for mean $PM_{2.5}$ associated with group-specific respiratory mortality in the Mexico City Metropolitan Area (2004-2019) for infants, children and adolescents.

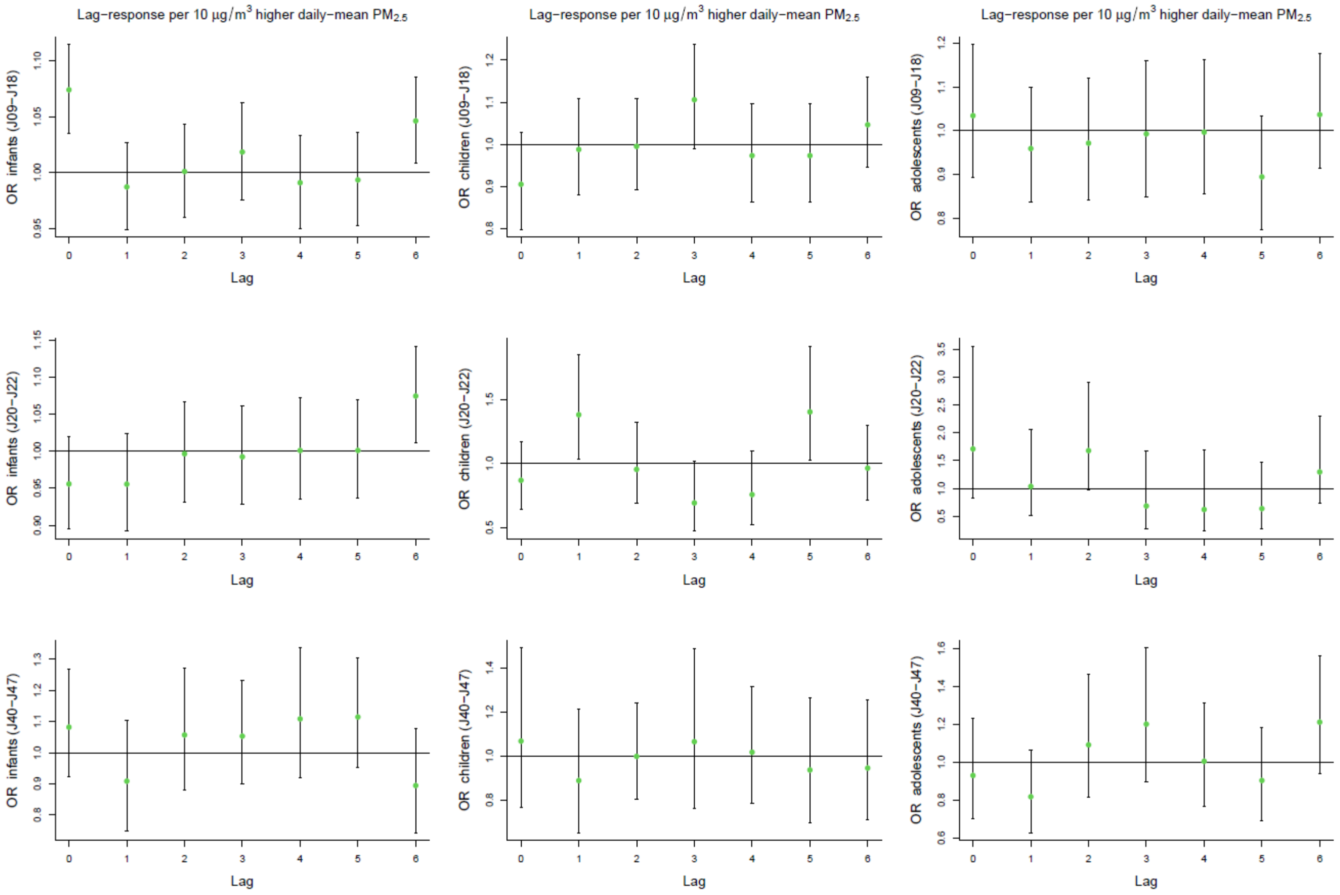

Influenza and pneumonia (ICD-10 codes: J09-J18), acute-lower respiratory infections (ICD-10 codes: J20-J22), and chronic-lower respiratory diseases (ICD-10 codes: J40-J47)

Neonate's plots were not included given the imprecise effect size of the associations.

Figure S3. Lag - response plots for max-1hr $PM_{2.5}$ and non-accidental mortality in the Mexico City Metropolitan Area (2011-2019) stratified by age group.

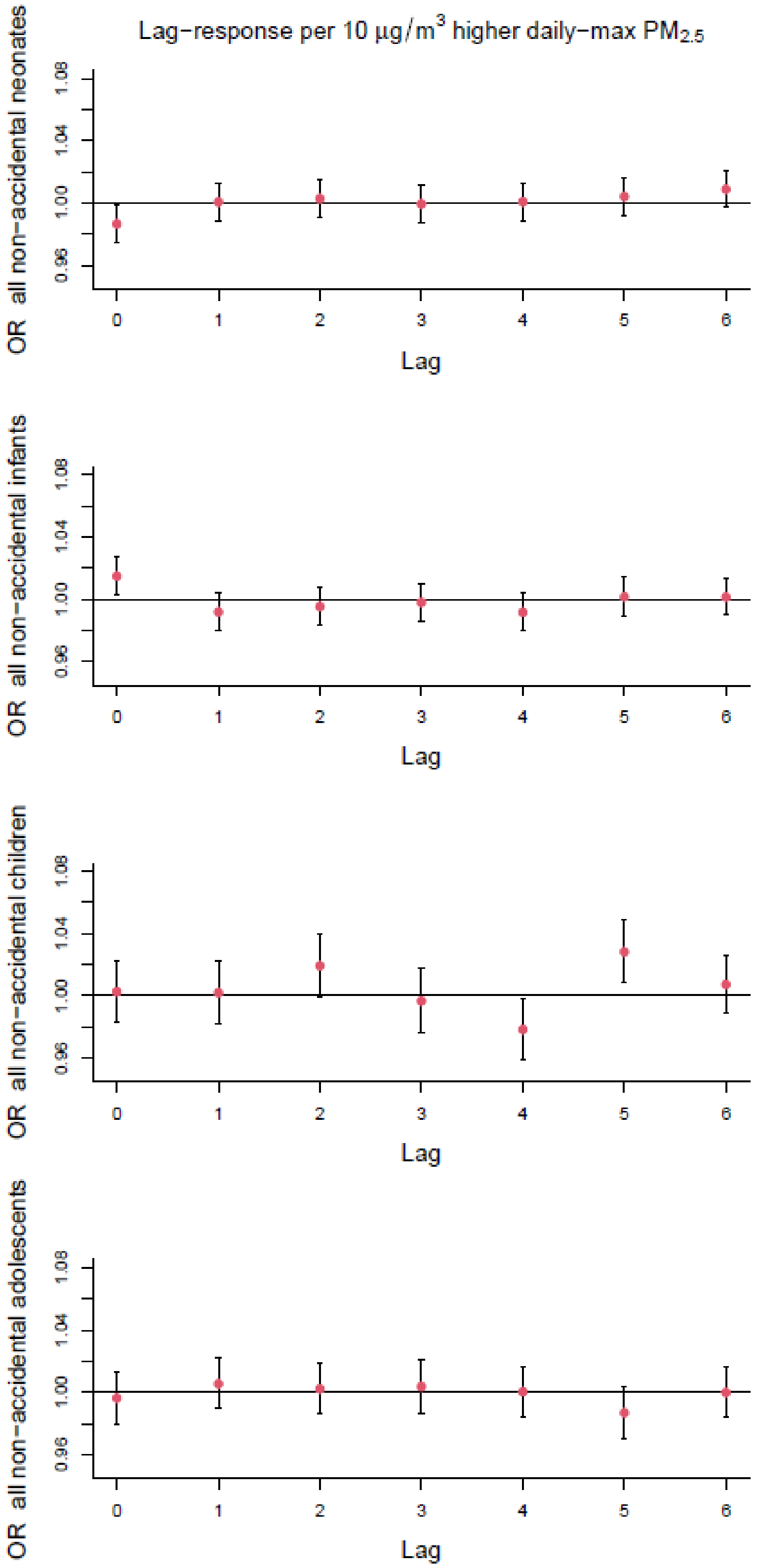

Figure S4. Lag - response plots for max-1hr $PM_{2.5}$ and respiratory mortality in the Mexico City Metropolitan Area (2011-2019) stratified by age group.

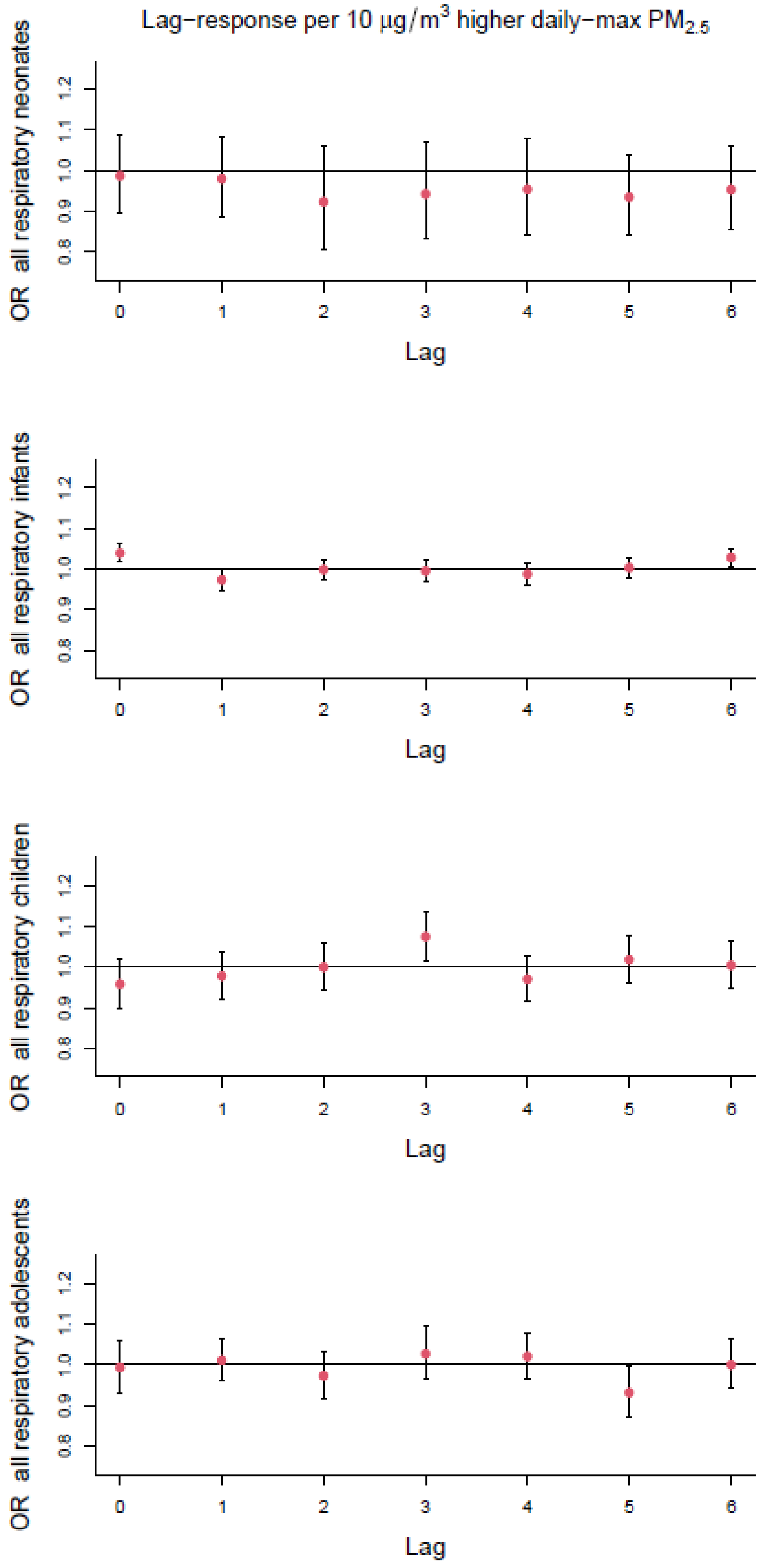

Figure S5. Lag - response plots for max-1hr $PM_{2.5}$ and group-specific respiratory mortality in the Mexico City Metropolitan Area (2011-2019) for infants and children.

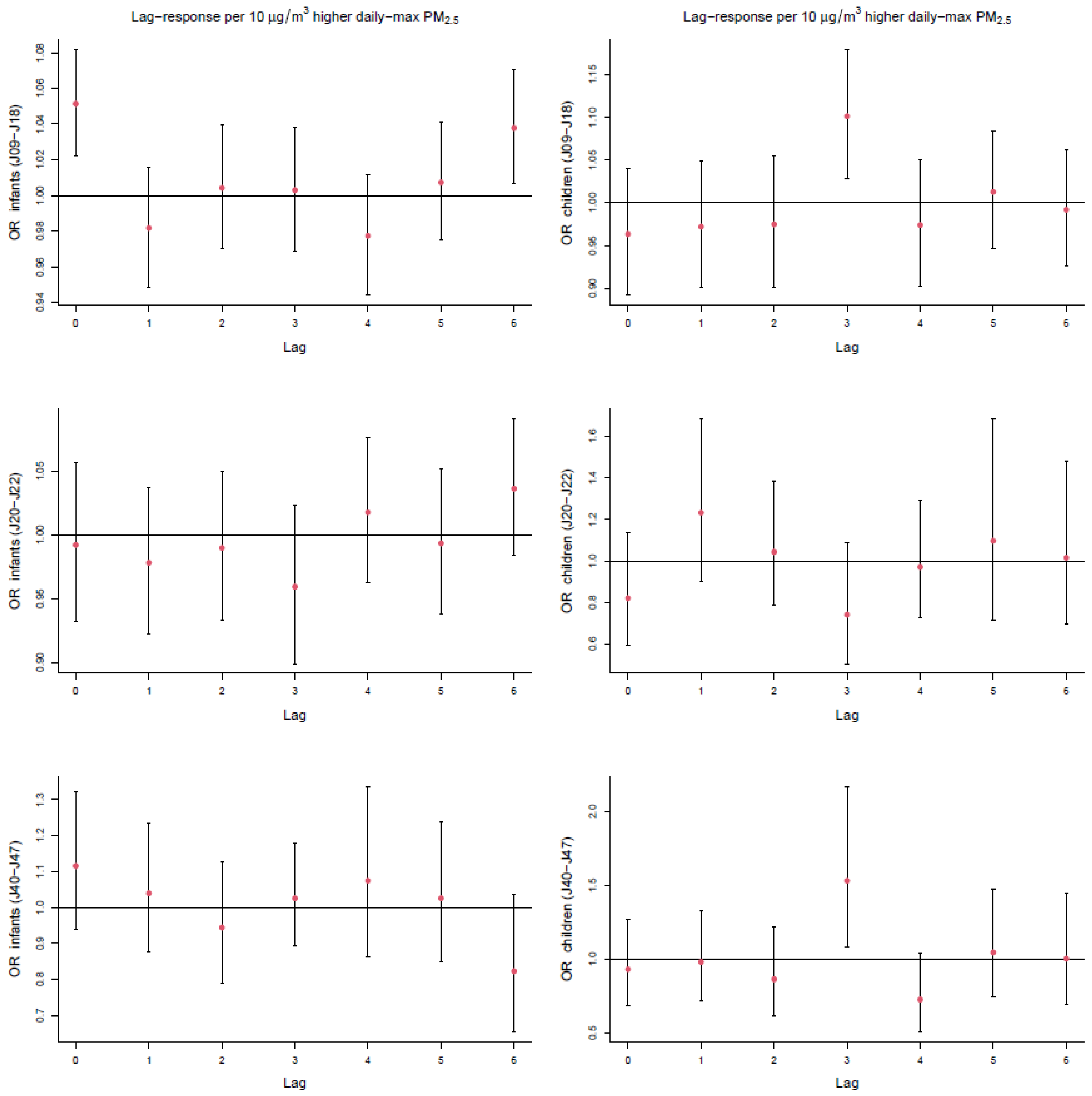

Influenza and pneumonia (ICD-10 codes: J09-J18), acute-lower respiratory infections (ICD-10 codes: J20-J22), and chronic-lower respiratory diseases (ICD-10 codes: J40-J47)

Figure S6. Lag - response plots for max-1hr $PM_{2.5}$ and respiratory mortality in the Mexico City Metropolitan Area (2004-2019) stratified by season.

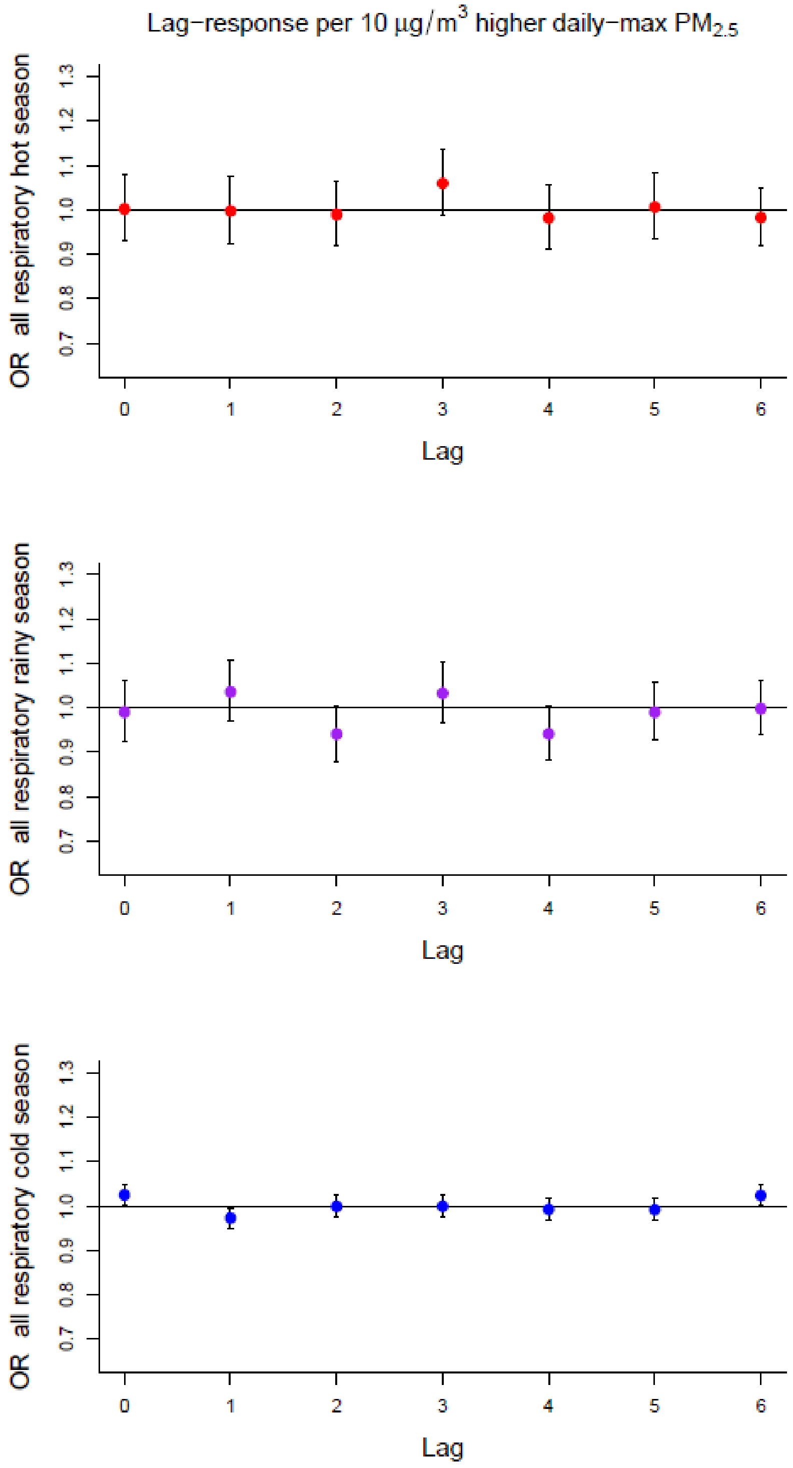

Hot season: March to May; Rainy season: June to October; Cold season: November to February.

Figure S7. Lag -response plots between mean $PM_{2.5}$ exposure with respiratory mortality in the Mexico City Metropolitan Area (2004 - 2019), stratified by sex and age group.

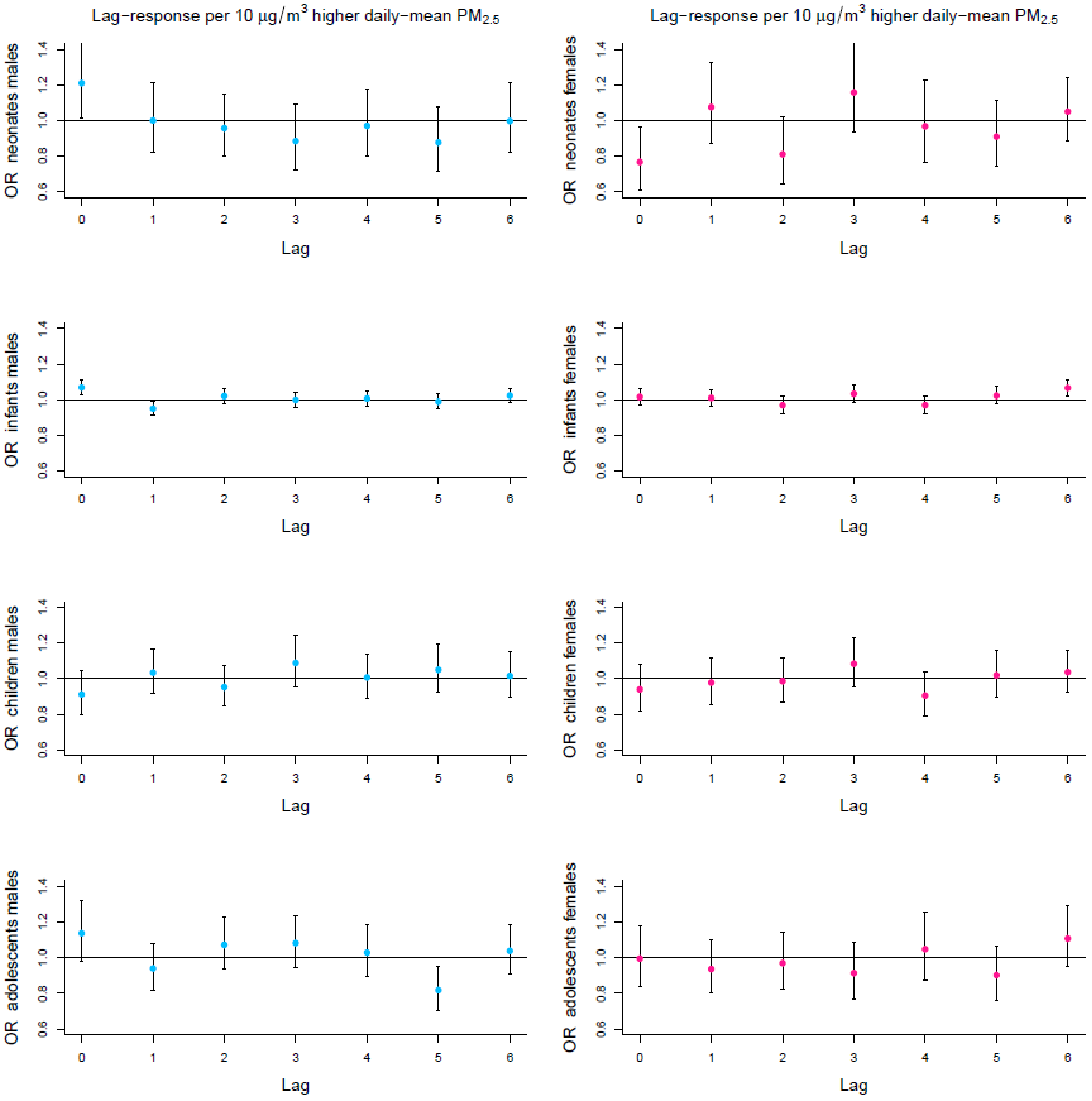

Supplement: Supplementary file 1 [file ee9-9-e408-s001.pdf]
